# The Rebuilding of the London Hospital

**Published:** 1906-02-10

**Authors:** 


### THE REBUILDING OF THE LONDON HOSPITAL.

A Correspondent has written the following account of this work, which was published in the *Times* of the 5th inst.:—

"At a time when the public is being urged from so many quarters to support charitable schemes which, from the very vastness of the proposals, raise some doubts as to their practicability, it is refreshing to turn to the successful conclusion of one of the greatest and most useful. Within the last few weeks the reconstruction of the largest general hospital in England has been completed, at a cost of over £430,000. This of course means that it has practically been rebuilt. The London Hospital now presents a remarkable example of perfection in so far as a well-built structure, part of which dates from 1752, can ever compete with new buildings erected regardless of the value of land. Of the great sum which has been expended on the work at least £330,000 has been subscribed for the direct purpose in question by the public. The remaining £100,000 has been taken from the invested funds of the institution, as the £5,000 a year which King Edward's Hospital Fund has regularly paid to the hospital since 1897 has been considered to be sufficient security for the loss of interest which would otherwise be required for maintenance. As a matter of fact this £100,000 was all that the management intended to spend when the alterations were originally decided upon. But the actual and useful expenditure of £430,000, rendered possible chiefly by the exertions of the Chairman, the Hon. Sydney Holland, shows how very urgent the original needs must have been.

"The state of the London Hospital eight years ago amounted to a scandal, if that expression may properly be employed in connection with a charity which after all is purely voluntary. At that time it had 780 beds and but one operating theatre—a fact in itself sufficiently shocking to anyone acquainted with hospital work. The pressure on this single theatre was such that the unfortunatee patients had to wait their turn in a fashion which amounted to absolute cruelty. Those who know what being prepared for an operation means, both physically and mentally, will understand the suffering occasioned to patients who were prepared as often as three or four, and in one recorded case seven, times in vain. The London Hospital to-day has eight operating theatres, five of which are in daily use, the other three being required not less than four days a week. The present number of beds in the hospital being 937, the theatre accommodation is one to 117 beds of all classes, as against one to 780 beds in 1897. The medical staff, however, were working under well-nigh impossible conditions in many respects besides those relating to operations. The out-patient department was totally inadequate to deal with the 161,000 individual out-patients who then sought relief, a number which has since increased to 206,000. There were no proper seclusion and isolation wards. The ophthalmic cases were accommodated in a damp basement below the ground level, and the arrangements for children were such that the constant outbreaks of infectious disease paralysed the whole work. In other important departments of this huge establishment the necessity for rearrangement was equally felt. The construction of the kitchen was so bad that no cook would stay. The system of lighting throughout the building was gas. The resident medical staff had no sitting-rooms, and the night nurses, for want of beds of their own, were sleeping in the day nurses' beds. All these shortcomings have now been remedied; and whatever else the East End may have to complain of, it is at least fortunate in its hospital.

"The cost of doing things on such a scale as is necessary where the daily service includes the treatment of over 800 sick people in bed and some 1,400 patients attending, either as new or old cases, in the out-patient department, runs into large figures. The out-patient department alone cost the London Hospital £83,000, the isolation block £29,000, the nurses' home £48,000. Furnishing amounted to £23,000, and the installation of the electric light to £13,000. Large sums were also spent in raising the old buildings to provide the extra accommodation required. It must, moreover, be remembered that the entire work was carried out without closing a single bed. This not only considerably increased the expense, but from time to time caused much inconvenience to the builders. Hammering had to be stopped every day while the doctors went their rounds. In one ward a floor was put down with screws instead of nails, at an increased cost of £40, in order to spare the patients below. The entire work of a wing had on one occasion to be stopped for the sake of a single patient, and temporary buildings alone cost nearly £5,000. In one way and another the great total of £430,590 has not only been expended, but paid for; and, whatever room there may be for criticism as to details, the work itself remains as an asset, the value of which to the poor of East London can scarcely be over-estimated.

"This is not the occasion for criticism so much as for congratulation. A great work has been accomplished in a comparatively quiet way; for, although Mr. Holland's ordinary methods of raising money for his hospital are perhaps the criterion of vigour, there were no preliminary blasts such as we are accustomed to expect when much smaller charitable undertakings are taken in hand. It would indeed be surprising if the expenditure of so great a sum were made without any mistakes either of principle or of detail. It may well be that critics of the present indiscriminate system of affording relief to out-patients may doubt whether provision has not been made for many more patients than in the future will be allowed to partake of free medical treatment. A time may come when the middle classes will ask whether it is really necessary that they should provide for the lower classes treatment so perfect and so far in excess of anything they can purchase for themselves. But that time has not come yet. Whether it ever comes or not, there is much to be said for a system which in effect guarantees to the working classes in great cities that, whatever other troubles they may have to contend against, they shall in time of sickness receive attention which, from some points of view, is scarcely obtainable by a millionaire."

